# Supplementary material for: Are carbon emissions trading and green financial instruments synergistic? -Comprehensive quantitative research based on content analysis
Source: PLoS One. 2024 Mar 7;19(3):e0298601. doi: 10.1371/journal.pone.0298601 (PMC10919601; doi:10.1371/journal.pone.0298601)
Supplement: S1 Data — (ZIP) [file pone.0298601.s001.zip › Data/地级市Z_F工作报告.zip!/║┌┴·╜¡/─╡╡ñ╜¡╩╨/└┤╫╘╣1⁄2╓┌║┼í╛┬φ┐╦╩2╛▌═°í┐_2009.docx]

各位代表：

　　现在，我代表市人民政府向大会作工作报告，请予审议，并请各位列席人员提出意见。

　　一、2008年工作回顾

　　过去一年，市政府在省委、省政府和市委坚强领导下，在市人大、市政协监督支持下，紧紧依靠全市上下共同努力，开拓创新，攻坚克难，圆满完成了市十四届人大二次会议确定的目标任务，保持了追赶型跨越式发展的良好态势。

　　（一）经济继续保持快速增长。“双五”目标如期完成，预计（下同）地区生产总值实现500亿元，增长14%。全口径财政收入完成55亿元，增长27.1%（不含绥芬河完成44.9亿元，增长35%），其中市本级财政收入21.8亿元，增长32.4%。财政支出完成75.3亿元，增长27.4%。争取财政和社保资金51亿元，增长近一倍。投资保持快速增长，全社会固定资产投资完成210亿元，增长35%。粮食获特大丰收，总产量达到38亿斤，农村经济总收入达到203亿元，增长16.7%。工业经济上升到近十年最好水平，规模以上工业企业实现增加值65.8亿元、销售收入240亿元、税金12.6亿元，分别增长24.7%、27%和22%。内外贸易活跃，社会消费品零售总额实现197亿元，增长24.5%；外贸进出口总额达到96.8亿美元，增长26.7%。城乡居民收入与经济同步增长，城镇居民人均可支配收入10700元，增长16%；农民人均纯收入6555元，增长17.7%，连续六年居全省首位。

　　（二）项目和工业园区建设成效显著。扎实开展“准备之冬”、“动工之春”、“百日攻坚”和“机遇之夏”活动，全市施工项目426个，其中当年新开工296个，完成投资158.6亿元，新谋划投资千万元以上项目365个，均创历史新高。园区建设如火如荼，沿301国道对俄经济带七个园区新上项目78个，其中市开发区新开工项目21个，虚拟镭射键盘等8个项目竣工投产，建成5万平方米标准厂房和园区科技孵化器，“双十六”万吨树脂和烧碱等5个项目加快建设。循环经济试点取得进展，化工污水处理厂和粉煤灰再利用项目获国家支持。海林园区形成“一区多园”格局，穆棱园区成为吸引粤台投资重要载体，绥芬河海都机电城、东宁机械产品出口园区发挥了调整出口结构作用。20万吨电石等20个项目列入省煤电化基地重点项目，投资25.5亿元的二发电两台30万千瓦机组“上大压小”项目、投资10亿元的10万吨油页岩加工项目开工建设。桦林佳通轮胎产能达到600万套，富通空调形成产业集群，停产两年的炼油厂恢复生产，OTC焊接机等高新技术企业正在崛起。科技创新力度加大，辟建了国家级生产力促进中心和中小企业国际合作园，恒丰等6家企业列为省技术创新试点，我市荣获中国城市信息化推进奖。

　　（三）沿边开放与各项改革进入新阶段。建设沿边开放先导区成为省级战略，国家海关批准牡丹江市区为中俄陆海联运大通道启运地，口岸内移渐成现实。异地办照政策调整试点通过国家验收，绥芬河综合保税区项目进入国家审批程序，中俄韩物流园区合作项目正式签约。北京航班增加到每周10班，东宁口岸实现每周7天通关，通关效率进一步提高。成功举办木博会、中俄名优商品展销会、牛业发展大会、黑木耳节及中俄区域合作论坛、镜泊湖之夏和雪城旅游文化节等活动，办会规模、档次和对外影响力大幅提升。旅游发展基金增加到200万元，全年接待国内外旅游者619万人次，旅游业总收入40亿元，均增长20%。举办“韩国周”、俄罗斯“滨海边疆行”等活动，开展赴乌克兰、日本和东莞、上海等专题招商活动18次，全年招商引资到位资金162.8亿元，增长44.2%，实际直接利用外资1.1亿美元，增长44.5%。引进万向、广水电、国电电力等战略投资者，与大庆油田建立了地企合作关系，世茂、圣戈班集团8个区域总部落户我市。实施金融强市战略取得良好开端，出台了金融强市五年规划和相关扶持政策，开展打击恶意逃废银行债务专项行动，重奖对地方发展做出贡献的金融机构，创办全省首家村镇银行，响水米业进入上市辅导期，银行存贷款余额分别比年初增加85亿元和25亿元。国企改革取得新成果，俄罗斯东华集团收购水泥厂，牡纺政策性破产终结，桦林集团进入破产程序。组建了镜泊湖旅游集团和医药产业协会。事业单位撤并转机构23个，合并市京剧团等团体，成立市文化艺术中心，市环卫处率先实现从“以费养人”向“以费养事”过渡。

　　（四）新农村建设向更高水平迈进。特色农业不断发育，农业结构更趋合理，食用菌发展到15.3亿袋(块)，经济作物达到70万亩，畜禽养殖业向规模化、标准化发展，一品村发展到440个，新引进南京雨润等龙头企业，规模以上企业达到85家，新转移农村劳动力1.1万人，畜牧业、食用菌、经济作物和劳动力转移收入达到农民人均纯收入的61%。落实惠农政策，发放“四补”资金3.6亿元。实施农业“百园”工程，创办20多个科技示范、休闲观光园区，中俄农业高新技术园区、农产品出口加工园区、宁安农场现代农业示范园区加快建设。境外耕种土地150万亩，地产农产品出口占全省70％以上。累计流转土地132万亩，宁安林权改革敲响第一槌，专业合作组织发展到410个，启动34个共建项目。新建农村公路2413公里、安全饮水工程112处，改造泥草房17.7万平方米，首批65个省级试点村通过验收，自来水普及率、砖瓦化率、有线电视入户率等指标远远高于全省平均值。

　　（五）城乡面貌发生可喜变化。全年城乡建设投入85.9亿元，是上年的2.1倍。鹤大线杏山至省界段、宁安至杏山段一级公路及雪乡旅游公路交付使用。整修市区街路76条，市开发区南区兴凯湖路和北区裕民路等4条街路竣工通车。城市规划展示馆、北山五星级宾馆等工程主体完工，东四跨江桥二期、大湾水利枢纽、三间房水电站等工程开工建设，东四跨江桥一期获国家建筑工程鲁班奖。首个政府主导、市区最大的棚户区改造项目曙光新城一期工程进展顺利，全市改造棚户区97.2万平方米，其中市区51.8万平方米。深入实施城乡洁净工程，粉刷了市区6条主街路两侧的289栋旧楼房，背街小巷及庭院亮化率达到90%以上，“一河三溪六湖”治理和人民公园改造工程完成年度任务。城市管理力度加大，整顿交通秩序和以牌匾广告等为重点的城市综合环境治理工作取得重大成效。生态和环境保护得到加强，治理水土流失38.2万亩，绿化造林5.75万亩，新增绿地6.15万平方米。海林污水处理厂投入运营，西水源地工程等10个松花江流域治理项目及5县（市）污水处理厂开工建设。锅炉并网107万平方米，市区空气质量达到二级标准，年度减排任务全面完成。

　　（六）民生工程和社会事业进一步惠及群众。50件利民实事除桦林供水工程等6项需跨年度推进外，其余全部完成。率先在全省将小额贷款额度提高到５万元、期限延长到4年，投入1500余万元开发公益性岗位，新增就业6.3万人，城镇登记失业率控制在3.7％以内。率先在全省将社保补贴范围扩大到集体企业失业人员，失业保险金标准提高到260元，城乡低保标准分别提高到月人均218元和年人均900元，离休干部和建国前老工人护理费人均月增加540元，退休人员养老金标准提高21.3％。新农合参合率达到95.2%，城镇居民医疗保险开始运行，社区卫生机构覆盖面达到95%，基本医疗保险和大额医疗最高支付限额提高到3万元和17万元。6所农村敬老院改扩建工程完工，启动了城市居家养老工程。落实廉租住房300套，为4700户低收入家庭提供廉租房货币补贴，为低保对象发放物价补贴3400万元，资助特困学生8300名。向四川地震灾区捐助款物4451万元。实施物价调控，开展打假活动，保持了市场基本稳定。免除城市义务教育阶段学生学杂费，完成了第九中学等3所学校教学楼改扩建工程。设立文化发展基金，自主创作的动漫剧《青果校园》在央视播出，评剧《金桂兰》获国家评剧节五项大奖，CCTV“激情广场大家唱”走进牡丹江。我市被确定为国家人口和计划生育工作示范市，穆棱进入全国少数民族“兴边富民”重点县。积极开展“全民健身与奥运同行”活动，竞技体育获国内外比赛金牌38枚。

　　（七）政府自身建设得到加强。完善了政府新闻发言人等制度，推进政务公开由信息公开向办事制度公开延伸，审核确认行政权力2183项，取消64项。整合加强了督办机构，精简临时、协调机构122个。认真贯彻突发事件应对法，狠抓安全稳定，突发公共事件下降25%，生产事故、治安刑事案件呈下降趋势，我市成为国家安全生产应急预案管理试点。开展市、县（市）区领导干部“大接访”活动，企办校拖欠教师工资等信访难点问题得到解决。坚持向市人大及其常委会报告工作和向市政协通报情况，办理人大代表建议、批评和意见62件，政协委员提案240件。深入开展“治庸治懒”活动，机关效能建设和依法行政得到加强。此外，政府的其他各项工作也都取得了新进展。

　　各位代表，过去一年的成绩，是上级党委、政府高度重视、关心和支持的结果，是市委正确领导、市人大和市政协监督支持，全市各方面同心协力、顽强拼搏的结果，是驻牡部队、中省直部门以及外来投资者倾力奉献的结果。我代表市政府，向所有参与、支持和关心牡丹江发展的各界朋友，表示衷心的感谢！

　　在30年改革开放进程中，我市经济建设和社会事业取得了辉煌成就，人民群众生活水平得到较大提高。2008年，全市地区生产总值、全口径财政收入、城镇居民人均可支配收入、农民人均纯收入分别是1978年的46.6倍、25倍、28.5倍和54.6倍。站在建设“大牡丹江、新牡丹江”和更高水平小康社会的新起点上，我们清醒认识到，虽然我市发展步伐加快，但与发达地区的差距仍然很大；我市三次产业正在恢复扩张，但产业结构优化升级的任务相当艰巨；我市沿边开放地位重要，但中心城市和口岸的辐射带动作用发挥得不够；我市城市功能尚不完善，城乡一体化进程不快；政府债务负担沉重，仅今年到期应偿还的本金和利息就达7.9亿元；公用事业历史欠账较多，很多民生问题亟待解决；经济发展环境不尽人意，公务员提高素质和转变作风迫在眉睫。与此同时，一场百年不遇的国际金融危机正加速蔓延，市场发生急剧变化。一些生产企业受大环境影响，销售开始受阻，严重的已经减产、停产；去年对俄贸易同比下降7.15%；小额贸易双倍抵扣政策调整，导致财政压力加大。“危”中见“机”，国家为应对金融危机出台了扩大内需、保持经济平稳较快发展的一系列政策措施，省委经济工作会议作出加快建设“八大经济区”重大决策，我市在四个经济区中榜上有名。危机应对得力，可使影响最小；机遇把握得法，可使效应最大。我们要辨证地看待当前形势，毫不动摇地加快发展，以对历史高度负责的态度和“面向东北亚、领跑黑龙江”的豪情，重新审视牡丹江，重新定位牡丹江，重新规划和建设牡丹江，推动牡丹江融入全国、走向世界。

　　二、2009年工作任务

　　今年是我市追赶型跨越式发展的关键一年，也是新中国建国60周年大庆之年。我们要把应对金融危机、抢抓重大机遇、保持快速增长作为头等大事来抓，落实市委十届六次全会提出的十项应对措施，帮助企业共渡难关，促进产品、产业结构优化升级；做好政策对接，积极向上争取项目和资金；完善配套政策，支持房地产开发和棚户区改造、廉租房保障；营造宽松环境，鼓励创业和就业；多渠道融资，千方百计筹措资金；超前谋划，加快公共项目建设；抢抓南方地区产业和资本转移机遇，扩大招商引资成果；突出载体建设，大力发展境内外园区，推动经济更好更快发展。市政府总的工作思路是：全面贯彻落实党的十七大和十七届三中全会精神，按照省委经济工作会议和市委十届六次全会部署，以科学发展观为统领，以改革创新、招商引资为动力，以保增长、扩内需、调结构、重民生、促和谐为目标，深入实施贸旅牵动、工业立市、金融强市战略，强化项目建设，加快财源振兴，全力打造黑龙江沿边开放先导区，继续保持全市追赶型跨越式发展的良好势头。围绕实现“双六”和财政收入三年翻番目标，全年主要预期指标安排为：地区生产总值增长14%，达到600亿元；全口径财政收入增长20%，达到66亿元；城镇固定资产投资增长40%以上，突破300亿元；农村经济总收入增长15%，达到233亿元；规模以上工业增加值、销售收入和税金分别增长20%、22%和20%；社会消费品零售总额增长16%，达到228亿元；外贸进出口总额增长16%，达到110亿美元；城镇居民人均可支配收入增长12%，达到11984元，农民人均纯收入增长12%，达到7342元；万元GDP综合能耗下降5.7%。我们要着重抓好七项工作。

　　（一）实施贸旅牵动战略，打造沿边开放先导区，不断提升沿边开放水平。

　　突出贸易和旅游牵动作用，着力建设东北亚地区加工中心、商贸中心、旅游中心、物流中心、会展中心，努力成为全省沿边开放的先导区、核心区，开展对俄、对韩、对日经贸科技合作的前沿区，内外互动，以外促内，以下促上，促进先导区由省级战略上升为国家战略，聘请国家发改委宏观研究院做好先导区总体规划，不断探索提升沿边开放新途径。

　　加快沿301国道对俄经济带建设。坚持整体规划、错位布局、功能协调、联动发展，探索以产业和项目需求划分区域功能，打破行政区域界限，最大限度地释放各园区发展潜能，重点规划发展好市开发区及海林、五林、下城子、绥阳等园区。理顺市开发区管理体制，用好国家循环经济试点等政策，一顶帽子大家戴，大力发展飞地经济。加快境内外园区标准厂房和配套设施建设，放大园区和企业品牌效应，引导企业和项目向园区集中，承接东莞等南方地区产业转移。着力构建集物流、会展、研发、加工为一体的木业产业集群，建设全国重要的木业博览中心、木材集散地、中国新兴地板之都。积极引进战略合作伙伴，大力开发适应俄罗斯市场需求的机电产品、轻工纺织、服装鞋帽、家具建材和有机食品，深化与俄罗斯在原油、重油、煤炭、电力等资源加工和利用上的合作，积极参与俄罗斯滨海边疆区的城市基础设施建设，拓展服务外包及电子信息、生物工程、新材料等高新技术领域的合作。抓住韩国本土企业外移、在华企业北移的机遇，积极引进韩资企业。

　　畅通国际经贸物流大通道。争取国家支持，完善口岸功能，进一步提升换运能力和通关效率，3年内口岸年过货能力突破1500万吨，年过客能力达到300万人次以上。促进内贸货物跨境运输常态运行，加快华晟国运二、三期建设，推进物流资源整合，规划建设农产品国际物流园区。开工建设总投资123亿元的牡绥铁路复线项目，配合做好哈牡城际铁路工程前期工作，为早日开工打好基础。打好公路建设“三年决战”，加快推进总投资55亿元的牡绥高速公路建设，开工建设鹤大线鸡西至牡丹江段等四条高速公路，确保牡宁段交付使用。完成机场跑道“盖被”工程，力争至海参崴等对俄航线复航，增加至韩国首尔航班，开通新的国际航线，做好机场搬迁前期工作。

　　加快辟建综合保税区和保税区配套区。争取绥芬河综合保税区尽快获批，做好保税区及配套区发展规划，加快建设，促进保税功能向牡丹江全境延伸。加强沟通协调，争取绥-波、东-波互市贸易区俄方一侧基础设施加快建设，充分发挥两个互市贸易区的政策效应，保持对外贸易平稳增长。

　　扩大人文交流。落实与俄罗斯滨海边疆区各市（区）交流计划，广泛开展对俄、对韩及对日人文交流活动。举办牡丹江国际大学生马拉松邀请赛，继续开展对俄教师交流及对韩公务员交流活动，进一步拓展在教育、医疗、文化、体育等领域的交流与合作。

　　努力做强旅游产业。聘请权威机构，编制镜泊湖渤海国旅游集合区发展规划。创新旅游管理体制，整合景区资源，发挥整体效应。推进镜泊湖温泉度假村、三道关国家森林公园度假区等项目建设，包装开发东宁三岔口朝鲜民族风情园等旅游项目，大力开发旅游文化和旅游商品。将旅游发展基金增加到300万元，加大在央视等主流媒体宣传力度，强化我市“中国优秀旅游城市”影响力。以异地办照试点为契机，促进镜泊湖景区与周边景区互动，加强与大连、无锡、三亚等地以及俄、韩等国旅行社合作，培育边境游及跨区跨国旅游热线。全年接待境内外游客712万人次，旅游业总收入46亿元，均增长15%。

　　围绕发展通道经济、临岸经济和发挥中心城市作用，大力发展现代服务业。举办好第五届木博会、第四届黑木耳节及镜泊湖之夏和雪城旅游文化节等活动，探索组建会展服务公司。规范发展会计、审计、信息、咨询、律师等中介机构。以中韩电子商务平台为突破口，培育发展对韩服务外包基地。引导扶持发展东安中央商务区、朝鲜民族风情街及光华汽贸一条街等特色街区，培育新型业态，繁荣活跃市场，积极扩大内需，促进消费升级。

　　（二）强化工业立市战略，突出抓好大项目建设，切实增强经济发展后劲。

　　调整优化工业结构。坚持走新型工业化道路，大力培育和做大做强资源转化、能源开发、进出口加工和高新技术产业。扶持发展龙睿系列、顺达电石等煤化工企业，积极开发后续产品及终端产品，做大重化工产业。高标准抓好硅产业园规划，开发有机硅系列产品。加强与大庆油田合作，做大石油钻具产业集群。扶持桦林佳通、富通等企业发展，争取大宇汽车等企业进驻，壮大汽车配套产业。支持北车集团在我市扩张产能，打造全国铁路载重机车零配件加工出口基地。用好省级爱民北药园区品牌，加速医药企业及资源整合，辟建实体医药园区，探索组建医药销售和药品加工外包服务集团，推动医药产业发展。通过落实专项资金、实施资源集中统一开发等方式，巩固造纸、电力、木业等产业，发展煤层气、生物质发电等新能源产业。以壮大对俄出口加工基地为目标，改造提升机电产品、轻工纺织、农副产品加工等传统产业。引导企业追踪市场变化和最新科技成果，不断开发新产品、应用新技术。

　　狠抓大项目建设。加快推进200个市级重点大项目建设，推动恒丰特种纸、圣戈班碳化硅微粉、佳日热电联产两机两炉、友搏疏血通等技改项目加快建设，续建“双十六”万吨树脂和烧碱、10万吨油页岩加工、30万吨芳烃类化工等项目，确保汇瑞毛毯、大亚木业、宁安化肥扩建等项目投产运营，力争40万部虚拟镭射键盘、亿象中高密度板等项目达产达效。谋划推进5000吨维生素C等前期项目，争取尽早立项开工。切实做好节能减排工作，加快推进汽车配套、化工等行业治污工程，完成市区3个重点环保治理项目脱硫改造，限制并逐步取缔粘土砖生产，必保完成年度减排任务。

　　不失时机地做好项目生成和向上争取工作。注重把握我市在区域经济一体化发展中的地位，把握城市的功能和作用，把握我们自身的比较优势，把握当代科学技术发展新成果，潜心研究国家拉动内需的各项政策，重点在能源基础设施、水利设施、交通网络建设、经济适用房和廉租住房开发以及文教卫生体育等领域，选准切入点，增强对接度，新谋划生成投资千万元以上项目500个以上，最大可能地获得国家、省及金融部门支持。

　　进一步加大招商引资力度。完善招商体制，创新招商形式和考核体系，调动各方面的招商积极性。以对俄合作为“王牌”，巩固与现有战略投资者合作成果，加强高层对接，开展小分队专题招商，促进俄油深加工、林浆纸一体化、抗肿瘤药物等重大项目落地，推动香港龙睿、大庆油田、大亚集团、欧米亚等大集团在我市设立区域总部，促进以商招商，广泛吸引新的战略投资者。组织开展长三角、珠三角及对台招商，举办“日本活动周”等大型推介活动，推动引项目、引企业向引产业发展，全年招商引资到位资金211亿元，增长30%。

　　（三）推进金融强市战略，加快改革创新步伐，为经济社会发展提供源动力。

　　推动金融业与地方发展相融合。继续开展打击恶意逃废银行债务活动，不断改善金融生态环境。进一步完善对金融机构的奖励措施，搞好银企对接，引导银行机构创新金融品种，增加对本地企业和项目的贷款额度。做大国有担保机构，规范完善扶强民营担保公司，引入农业保险公司。深化农村金融改革，继续发展村镇银行，完善农户配套担保贷款模式，建立全省首家小额贷款公司，争取列入全国农村金融体系建设试点，做好政策性农业保险扩面工作。争取发行信托产品、企业债券，推进城市商业银行增资扩股，积极引进股份制商业银行和外资银行，促成韩国韩亚投资银行与我市开展金融合作。强化对企业改制上市、并购重组、项目融资、资产管理等方面的服务，推进响水米业、天合石油机械等企业上市进程。

　　进一步改革完善投融资体制。坚持融资、解债和资产运营同步推进，整合市城投集团、广兴国有资产经营公司，建立“借、用、管、还”一体和“责、权、利”统一的投融资机制。抓好省开行牡丹江金融驻贷机构建设，做实县域经济及中小企业融资平台。加强与进出口银行等政策性银行及长城、信达等资产经营公司合作，通过整合资产、回购债权、拍卖转让等途径，盘活国有资产，化解历史债务。抓好城市经营，在置换土地推进棚户区改造、以TOT方式运作自来水公司、转让资产资源经营权等方面取得新突破。

　　全力促进财源振兴。实施市本级财源振兴规划，突出抓好市本级财源建设。切实帮助企业解决生产经营困难，扶持骨干企业、成长型企业发展壮大，培育高新技术和现代服务业等新兴产业，巩固现有财源，发展后续财源。实施在地统计，发挥经济普查对财源建设的基础性作用。细化发展飞地经济、总部经济的措施办法，完善经济发展特别奖和共享收入激励机制，理顺市直与城区财政体制，整顿税收秩序，避免税源流失。强化财税征管手段，对重点税源实施精细化管理，推进非税收入收缴制度改革，提高外资企业、第三产业和非税收入对财政的贡献率。增强过紧日子思想，严控财政供养人口增长，加强预算外资金管理，缩减弹性支出。强化财政绩效评价考核，提高财政资金使用效益。

　　注重体制机制的根本性作用，深化重点领域改革，增强发展活力。妥善解决国企改制遗留问题，继续做好企业政策性破产工作，彻底解决破产企业退休人员医疗保险问题，推进炼油厂改制重组，完成桦林集团破产。从上新项目入手，广泛吸引多元投资，大力发展股份制经济和民营经济。推进事业单位资源整合，理顺事业单位与政府、企业、社团关系。深化事业单位人事制度改革，实行人员“凡进必考”制度。实施优秀村支书公职化管理。按照国家和省部署，适时启动医疗卫生体制改革。

　　强化科技和人才的保障作用。牡丹江追赶跨越最终要依靠科技和人才做支撑。完善科技信息网，加强公共研发、公共技术、公共检测、公共信息等开放平台建设。强化自主创新投入机制，组织企业开展科技创新，推动质量兴市，培育名牌产品，创建名牌企业。建立产学研合作机制，加强与哈工大及俄罗斯等国内外高等院校、科研机构合作共建，促进科研成果转化。突出引资与引智相结合，完善人才政策，增加和用好人才发展基金，加快市人才市场建设，重点引进国际贸易、金融证券以及各行业急需的专业技术和管理人才，大力培养技术工人和实用型人才。

　　（四）以农民增收为核心，统筹城乡发展，扎实推进社会主义新农村建设。

　　提升壮大特色优势产业。加快养殖基地建设，扶持皓月、鑫鹏、正大等龙头企业发展。做大食用菌市场，增强雨润、三通等加工企业带动能力，扩大食用菌栽培规模，栽培总量达到17亿袋（块），推进食用菌产业提档升级，提升“中国食用菌之城”影响力。围绕瓜果菜、烤晒烟等高效经济作物，率先推行高产栽培、大机械耕作、适度规模经营新模式，建设沿201、301国道和“两湖”周边10个示范带（区）。培育特色养殖业和名优水产业，抓好“百村万户山里人家”乡村旅游试点。推进市级农业“三大园区”和“百园工程”建设，新命名挂牌园区40个。强化农产品安全质量监管，扩大市场营销，叫响一批消费者信得过的“牡丹江特产”。深入实施农民培训“阳光工程”，提高农民生产和创业就业能力，新转移农村劳动力8000人。启动对俄农业合作提升工程，拓宽农产品出口通道，壮大境外种养业和农产品加工业，促进东宁县率先实现农民人均纯收入过万元。

　　加强农村基础设施建设。抢抓新一轮农村改革发展和全省实施千亿斤粮食产能工程机遇，增加农村建设投入。实施水利化工程，新建6个千亩标准化节水灌溉示范区，实施9座水库除险加固工程，治理水土流失26万亩。实施农机化工程，落实“农机下乡”政策，推广适合我市特点的农业机械，农业综合机械化程度达到83%。按照新标准加快新农村建设，新建通村公路350公里，行政村通村公路硬质路面通达率达到92%，改造泥草房15.2万平方米，新建饮水安全工程120处、浴池100个、卫生厕所3000个，推广沼气、地能等新型能源，新建“康居乡镇”5个、“康居新村”36个，推进“每县一乡、每乡一村”新农村建设精品工程。开展“万村千乡市场”和“家电下乡”活动，改善农民生产生活条件。

　　稳定和完善农村基本经营制度。扩大土地承包经营权流转市场建设试点范围，全市1/3乡镇建立规范的流转市场。鼓励农民以多种形式流转土地承包经营权，支持种植大户、龙头企业、经合组织牵头组建土地合作社，促进土地集约化经营。深化宁安国有林权制度配套改革，启动集体林权制度改革。壮大农民经合组织和经纪人队伍，提高信息化服务水平。深化动植物疫病防控体系改革，提高动植物防疫水平。发挥农业科研院所作用，加快农业科技成果转化和技术推广。

　　推进城乡一体化发展。围绕建设“大牡丹江”，进一步完善资源整合、区域联动发展机制，完成场镇、局镇、企镇共建和城区、园区、景区、口岸带动“七种模式”建设规划，开展乡镇经济和社会综合实力评价工作，加快特色乡镇发展。推进共建城镇扩权试点，破解户籍、就业、就学、医疗、社保等制度瓶颈。大力发展县域经济，放大绥芬河、东宁向内传导效应。通过政策和项目扶持，推动“突破林口”战略取得阶段性成效。促进各县（市）区联动发展，进一步提升在全省的位次。

　　（五）树立规划的权威性，加强城市管理，提升城市功能和品位。

　　高标准规划城市。强化规划的先导作用，聘请国内著名规划设计机构与我市合作，编制大牡丹江概念规划、城市景观规划，突出太平路、牡丹江两岸、城市出入口、机场、车站的景观规划，完成市区南北太平路城市中轴和沿江景观带“一轴一带”城市框架规划，继续推进区划调整，改造桥北，完善桥南，提升江南，打造东西延伸、南北贯通、一江居中、两岸同兴的城市格局。完善江南新城区和市区棚户区改造控详规划，把西三、东四跨江桥之间江南58万平方米核心区块打造成城市地标，突出建筑特色与周边环境的协调性，彰显城市个性。

　　不断改善城市基础条件。以打造宜居城市为目标，加快西十一跨江桥、东四跨江桥二期、大湾水利枢纽、牡丹江南岸堤防、三间房水电站、五花山水库等项目建设，力争林海水库等项目开工，筹建国际会展中心，确保北山五星级宾馆、城市规划展示馆等工程年内交付使用。加快棚户区改造步伐，确保曙光新城一期动迁户年底前回迁，启动“三溪”交汇处等其他区片改造，全市新改造棚户区156万平方米，其中市区达到60万平方米以上。出台相关政策，保障房地产市场快速健康有序发展。推行节能环保建筑和计量供热。动员社会力量，继续开展市政道路建设工程，整修街路巷道100条、人行道15万平方米。完成人民公园改造，建设江南江堤带状公园，续建水系治理截污管网4.4公里，开工建设污水处理厂二期和医疗废弃物处理中心，完成1号、3号泡二期工程和4号泡景观改造，两年内完成“一河三溪六湖”改造工程。市区新增管道燃气用户1万户，确保10个松花江流域治理项目及5县（市）污水处理厂竣工运营。开展“三年绿化牡丹江”行动，今年绿化造林25万亩。

　　理顺“大城管”体制。实行建管分开、政企分开、政事分开，促进园林绿化、环境卫生等公益性事业市场化，推动国家园林城、卫生城、环保模范城、文明城“四城同创”。落实环境集中整治三年规划，推进文明执法，强化细节管理，建设电子网络监控系统，解决铁路周边环境脏乱差问题。巩固整顿交通秩序成果，打击非法营运车辆，建立交警、运管、监察等部门联合管理体制，规范交通秩序。延伸公交线路，发展公共交通。取缔市建成区手烧锅炉及洗浴、餐饮、旅店业燃煤炉灶，锅炉并网50万平方米。培养市民公德意识和文明素养，壮大义工队伍，营造人民城市人民建、人民城市人民管的良好氛围。

　　规范完善小区物业管理。制定物业分级管理办法，建立质价相符的收费与服务体系，加强监督管理，提高物业企业服务水平，促进有实力的物业企业向专业化、规模化发展。开展弃管房调查，落实管理主体和责任。充分发挥业主大会和业主委员会的作用，融洽业主与物业管理部门关系。

　　（六）继续办好利民惠民实事，保障和改善民生，让广大群众共享发展成果。

　　多方筹措资金，扩大教育、卫生、文化、社保等公共财政支出，扎实做好民生保障工程，着力为群众办好22件实事。

　　努力实现群众安居乐业。关注民工回流和大中专毕业生就业形势严峻的新情况，强化市创业服务中心就业孵化作用，筹集资金5000万元，综合运用税收优惠、担保贷款、社保补贴、提供培训等措施，有针对性地鼓励创业、扶持就业，新增就业5.5万人。对“4050”人员、零就业家庭、城郊失地农民实施就业援助，开发公益性岗位2000个。加大保障性住房供给，解决200户优抚对象住房困难问题，为5000户低收入家庭提供廉租住房补贴。

　　提高社会保障和居民收入水平。抓好县级新型农村养老保险试点，实施被征地农民养老保险和建筑业农民工工伤保险制度，推进城镇居民基本医疗保险全覆盖，医疗保险报销比例提高5%。大力发展慈善事业，建成2个区级福利中心，开展城乡社区志愿服务、对口服务，推进帮扶、养老、助残工程，免费为400名贫困精神病患者提供治疗药物，免费发放残疾人辅助器具500件，免费为贫困弱势群体提供法律援助1200件，市区盲人免费乘坐公共汽车，投入800万元为1万户低保边缘职工送温暖，“爱心超市”救助困难群众3万人次。千方百计筹措资金，将养老金标准人均提高130元，城市低保标准月人均提高到240元、农村年人均提高到1000元，伤残军人抚恤金提高20%，“三属”补助提高15%，继续增加公务员和事业单位人员津贴补助。

　　坚持大教育发展战略，促进教育均衡发展。加快标准化学校建设，完成长安小学等3所中小学改扩建，改造农村初中校舍。深化校企协作，建设综合性公共实训基地。支持民办教育规范化发展，引进有影响力的民办教育集团。推进院市合作共建，促进高校与地方发展相融合。以“2009中国俄语年”为契机，建立俄语、韩语教学和留学生基地。实施名优校长、教师“双优”工程，提高师资水平。加强未成年人和大学生思想道德建设，健全外来人员子女、“留守儿童”教育保障机制，资助贫困学生就学，营造有利于青少年健康成长的社会环境。

　　提升居民健康指数。改扩建12所中心乡（镇）卫生院、20所中心乡（镇）计划生育服务站，促成农（林）场与地方医疗机构互为开放，提高卫生资源利用率。推进市二院、中医院改扩建工程，进一步改善医疗条件。完善“120”急救服务功能，提高社区卫生医疗服务质量，对低保对象、残疾人和60岁以上老年人免费体检。加强重大传染病预防控制和监测，确保公共卫生安全。施行计划生育新条例，强化优生优育措施，提高出生人口质量。

　　丰富居民文体生活。开展庆祝建国60周年系列活动，合作完成《镜泊湖保卫战》等影视剧拍摄。做好八女投江纪念馆布展工作，推进新华剧场改造，加快乡镇综合文化站建设。抓好第三次全国文物普查工作。整合经营性资源，做大出版、演艺娱乐等传统文化产业，发展动漫制作、文化创意等新兴文化产业。广泛开展全民健身活动，组建体育产业开发公司，承办大型体育赛事，举办好首届全市运动会。

　　按照把社区建成管理有序、服务完善、文明祥和的社会服务共同体要求，推进城乡新型社区建设。建立政府投入与社会投入、无偿服务与有偿服务、社会化服务与产业化服务相结合的社区投入和发展机制。加快基础设施建设，新建4个区级社区服务中心。推广10分钟便民服务圈经验，整合社区服务资源，大力发展社区经济，强化社区文化、教育、管理功能，创建和谐社区。抓好农村社区试点，探索城郊结合部、集镇中心村社区建设新模式。

　　(七)转变工作作风，提高行政效能，不断优化经济发展环境。

　　切实加强政府自身建设。坚持用科学发展观武装头脑，树立世界眼光，不断解放思想，围绕全市中心工作，增强项目意识、金融意识、口岸意识，提高抓发展和解决实际问题的能力。建立科学发展观评价指标体系，完善公务员管理考核机制，狠抓治庸治懒，加强诚信建设，创建机关建设品牌。认真贯彻国务院关于加强市县政府依法行政的决定，落实好行政问责和行政执法过错追究制度，提高依法行政水平。加快电子政务建设，推进政务公开，实现政务信息化。严格实行重大决策评估、公示和听证制度，定期向市人大报告工作和向市政协通报情况，及时办理人大代表议案、建议、批评、意见和政协委员提案，加强与民主党派、工商联、无党派人士和工会、共青团、妇联等人民团体沟通，努力做到科学决策、民主决策。毫不放松地抓好廉政建设，充分发挥监察、审计部门的作用，主动接受各方面监督，努力打造“阳光政府”。

　　牢固树立民本思想，强化公务员对企业、群众的服务。实施“两集中、两到位、三减少”改革，推进行政审批职能向一个科室集中、部门行政审批服务科室整建制向行政服务中心集中，保证审批职能归并和部门进驻中心到位，减程序、减时间、减费用，对重大投资项目实行审批绿色通道、领办代办和全方位跟踪服务制度，打造安商、亲商、富商的投资环境。整合市长热线、“城建110”，创建全天候的“12345”政府综合性公共服务热线。集中开展对行政执法部门以及垄断行业的行风检查评比活动，突出解决野蛮执法、收费过高和不合理收费等行为，发挥举报中心、行风热线和网络的监督作用，严肃查处影响企业正常生产经营、损害群众合法利益的不良行为。

　　健全完善维权、维稳、协调、创安机制，创建平安和谐牡丹江。推进“五五”普法，提高全社会法律素养和守法意识。加快社会信用体系建设，促进企业增强社会责任。加大市场秩序监管和联合执法力度，重点抓好食品、药品、农资、房地产及建筑开发等市场整治。高度重视和加强信访工作，妥善解决好动回迁、征地补偿、企业改制等信访问题。深入落实国家民族宗教政策，支持民族经济和民族事业发展。强化应急体系建设和日常管理，有效预防和控制各类突发事件。把安全工作当作“天字号”工程来抓，切实搞好煤矿和非煤矿山、道路交通、建筑工地、特种设备以及接送学生车辆等重点领域的安全防范，加强防汛、森防和消防工作，坚决避免重特大事故发生。强化社会治安综合治理，确保人民群众生命财产安全、社会安宁。

　　各位代表！今年我市发展的外部环境变数很大，而全市追赶跨越的步伐刻不容缓。我们要在市委正确领导下，解放思想，锐意进取，戮力同心，狠抓落实，为全面完成2009年的各项工作任务而努力奋斗，以优异成绩向新中国60周年华诞献礼！
